# Separating Leaf and Wood Points in Terrestrial Laser Scanning Data Using Multiple Optimal Scales

**DOI:** 10.3390/s19081852

**Published:** 2019-04-18

**Authors:** Junjie Zhou, Hongqiang Wei, Guiyun Zhou, Lihui Song

**Affiliations:** 1 Center for Information Geoscience, University of Electronic Science and Technology of China, Chengdu 611731, China; dear.zjj@hotmail.com; 2 School of Resources and Environment, University of Electronic Science and Technology of China, Chengdu 611731, China; cwnuweihongqiang@126.com (H.W.); lihuisong9610@163.com (L.S.); 3 State Key laboratory of Soil Erosion and Dryland Farming on the Loess Plateau, Institute of Soil and Water Conservation, Chinese Academy of Sciences, Yangling 712100, China

**Keywords:** multiple optimal scales, leaf and wood separation, terrestrial laser scanning, machine learning

## Abstract

The separation of leaf and wood points is an essential preprocessing step for extracting many of the parameters of a tree from terrestrial laser scanning data. The multi-scale method and the optimal scale method are two of the most widely used separation methods. In this study, we extend the optimal scale method to the multi-optimal-scale method, adaptively selecting multiple optimal scales for each point in the tree point cloud to increase the distinctiveness of extracted geometric features. Compared with the optimal scale method, our method achieves higher separation accuracy. Compared with the multi-scale method, our method achieves more stable separation accuracy with a limited number of optimal scales. The running time of our method is greatly reduced when the optimization strategy is applied.

## 1. Introduction

Tree characteristics are vital parameters for many environment applications, such as ecosystem productivity models, carbon dynamic and ecological studies, forest management, and disease and stress detection [1,2,3,4,5]. Manually obtaining many of these characteristics is a time-consuming process. Terrestrial laser scanning (TLS) has provided a revolutionary way to quantify individual tree characteristics, with detail, accuracy, and flexibility that satellite laser scanning and airborne laser scanning have not been able to match during the past two decades [6,7,8]. TLS data of an individual tree can be used to retrieve many tree parameters, including diameter at breast height, leaf area index, plant biomass, virtual projection, and gap fraction [1,9,10,11]. Some parameters of a tree, such as tree height, diameter at breast height, and crown width, can be extracted directly from raw TLS data. Retrieving other high-level tree parameters from TLS data requires the separation of leaf and wood points to improve accuracy and reduce complexity [12]. Béland et al. [13] distinguished leaf and wood points in TLS data before estimating three-dimensional (3D) leaf area distribution. To quantitatively remove the effects of the woody material in leaf area index estimates, Ma et al. [14] separated the TLS data into photosynthetic and non-photosynthetic points. Due to the natural heterogeneity and complexity of tree surfaces, separating leaf and wood points is technologically challenging [7,14,15,16].

Existing leaf and wood separation methods are either unsupervised or supervised. Unsupervised separation does not require training samples and the workload of the end users is limited. Béland et al. [17] used the contact frequency of co-registered TLS points from two or more scanning positions to separate leaf and wood points. Hakala et al. [18] designed a separation method that was based on the radiometric information of different wavelengths of a multi-wavelength laser scanner. Belton et al. [19] used geometric features and a Gaussian mixture model to cluster the points into leaves, trunk branches, or unknown. Béland et al. [13] identified an appropriate normalized radiometric information threshold value and then separated the leaf and wood points. Tao et al. [7] extracted the skeleton of a tree before separating the leaf and wood points. Li et al. [20] used the difference in the structures of different components of a tree to separate leaf and wood points [21]. Wang et al. [15] proposed a robust and dynamic point cloud segmentation routine to separate a tree point cloud into leaf and wood points. Ferrara et al. [22] introduced an approach that was based on the density-based spatial clustering of applications with noise. Xu et al. [23] provided an approach that used a bottom-up hierarchical clustering strategy to combine clusters belonging to non-photosynthetic components, which could also be used to separate leaf and wood points in a tree point cloud. 

The supervised separation methods require the input of training samples and can achieve higher accuracy and stability than unsupervised methods. Ma et al. [14] used the spatial distribution patterns of manually selected training points to train a Gaussian mixture model for leaf and wood point separation. Yun et al. [24] calculated the shape, normal vector distribution, and structure tensor of tree point cloud and used a support vector machine to separate leaf and wood points. In the above studies, only geometric features of tree point clouds were used and the radiometric information was not employed. Compared with the separation methods that employed only geometric features, methods that integrated radiometric and geometric features achieved better separation accuracy and robustness [25,26]. However, the radiometric values need an instrument specific radiometric calibration before they are used to separate leaf and wood points. The instrument specific radiometric calibration was a challenging process and more detailed studies were still needed for physical interpretation [27,28]. In practice, the geometric feature-based supervised methods were widely used to separate leaf and wood points for their accuracy, stability, adaptability, and expansibility.

For the geometric feature-based supervised separation method, the separation accuracy depends on the machine learning methods, selected scales, and geometric features. Wang et al. [16] examined four machine learning methods and 26 geometric features that were widely used in other separation tasks [29,30,31,32,33], finding that the random forest method and several geometric features could effectively separate leaf and wood points in TLS data. To assess the scale effect on the separation accuracy, Wei et al. [12] extracted the single-scale and multi-scale geometric features from the point clouds of two *Oak* trees and showed that the multi-scale geometric features improved the separation accuracy greatly. However, how to select the appropriate scales to calculate the geometric features was not discussed, which affected the separation accuracy greatly [14,24,26,31,32].

Three strategies can be used to select the appropriate scales. The first strategy heuristically selects one fixed scale for all points [14,24]. Although this strategy is simple and fast, it is data dependent. The second strategy, referred to as the optimal scale method hereafter, finds one optimal scale for each point [31,32,33,34,35]. The third strategy, referred to as the multi-scale method hereafter, randomly selects many fixed scales for all points [29,36]. The assumption of this method is that the surface of objects is heterogeneous and its distinctive properties are seldom defined at one specific scale [29].

In this study, we extend the optimal scale method to use multiple optimal scales, referred to as the multi-optimal-scale method hereafter. Compared with the optimal scale method, the multi-optimal-scale method achieves higher separation accuracy. Compared with the multi-scale method, the multi-optimal-scale method achieves more stable accuracy with a limited number of optimal scales.

The remainder of this paper is organized as follows. The experimental data are described in Section 2. In Section 3, the proposed multi-optimal-scale method is explained in detail, together with a brief description of the optimal scale and multi-scale method. Section 4 presents the experimental results and discussion of the proposed multi-optimal-scale method. We conclude our paper in Section 5.

## 2. Experimental Data 

**Figure 1:**
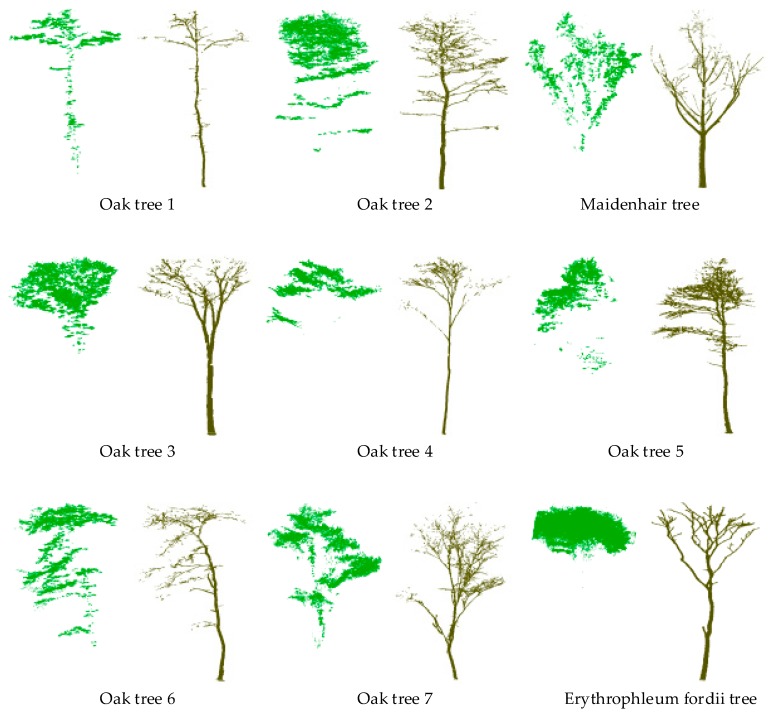
The manually separated leaf and wood of the trees.

Point clouds of nine trees, including one *Erythrophleum fordii* tree, one *Maidenhair* tree, and seven *Oak* trees, are used in our study. The *Erythrophleum fordii* tree was provided by Hackenberg et al. [10] and used in Wang et al. [15,16]. This data was acquired by a Z+F IMAGER 5010 from eight scan positions in October 2013 at Baiyun forest farm (106°45′ E, 26°06′ N), Guangxi Province, China. The *Maidenhair* tree was scanned by Leica ScanStation C10 from one scan position in May 2017 at the University of Electronic Science and Technology of China (104°07′ E, 30°07′ N), Sichuan Province, China. The seven *Oak* trees were scanned by Leica ScanStation P40 from four scan positions in April 2016 at Jigong Mountain National Nature Reserve (114°02′ E, 31°50′ N), Henan Province, China. *Oak* tree 1 and *Oak* tree 2 were also used in Wei et al. [12]. The *Erythrophleum fordii* tree was registered and preprocessed by Hackenberg et al. [10]. All other trees are preprocessed using the registration and edit modules of Leica Cyclone (Leica Cyclone 9.1.4, 2016) software. We manually separated the leaf and wood points of each tree to obtain the reference data to validate the accuracy of the models with the open-source software CloudCompare (CloudCompare 2.10-alpha, 2018). As this operation is based on the visual assessment of the tree point cloud, the quantitative evaluation results may be affected by the user interpretation slightly. In this study, we randomly select 10% of the aforementioned data as *core points* to reduce the processing time of our multi-optimal scale method (the details of the *core points* are shown in Section 3.4). The number of points, the average point density, the tree height, and the *core point* of each tree is listed in Table 1. The manually separated tree point clouds are shown in Figure 1.

## 3. Methods.

### 3.1. Scale Definition and Selection of Multiple Optimal Scales

Let $q\;=\;\left(x,\;y,\;z\right)\in R^{3}$ be a point in the 3D space. $Q\;=\;\{q_{i}\in R^{3}\;|\;i=1,\ldots ,N\}$ denotes the tree point cloud. For being able to describe the local 3D structure of a given point $q_{i}$ via geometric features and increase the distinctiveness of derived geometric features, the first step of our method was to select an appropriate scale definition and scale size for individual points. Two scale definitions are commonly used: Spherical scale and k nearest neighbor scale. The spherical scale requires a suitable radius, which is different for different tree point clouds [31]. The k nearest neighbor scale requires a parameter k, which is independent of the tree point clouds [32]. We employed k nearest neighbor scale in this study. The parameter k is a scale parameter and different values of k correspond to different scales.

Before selecting the scales, we needed to specify the set of candidate scales from which the selected scales are determined. Two strategies are commonly used to determine the candidate scales. In the first strategy, the candidate scales form a geometric sequence [34]. In the second strategy, the candidate scales form an arithmetic sequence [31,32]. The second strategy is simple but slightly increases computational load compared with the first strategy.

For each point $q_{i}$ in Q, a principal component analysis is applied to its k nearest neighbors [37]. The ordered eigenvalues resulting from the principal component analysis for point $q_{i}$ are $\lambda_{1}$, $\lambda_{2}$, and $\lambda_{3}$ ($\lambda_{1}\;\leq \;\lambda_{2}\;\leq \;\lambda_{3})$ and used to infer multiple optimal scales of this point. Let $\alpha_{i}=\lambda_{i}/\left(\lambda_{1}+\lambda_{2}+\lambda_{3}\right)$. $\alpha_{i}$ can be considered as the “probability” of a point being labelled as a 3D, 2D, or 1D structure [31]. The measure of the eigen-entropy can be defined via the Shannon entropy equation as

(1) $$E_{\alpha }=-\left(\alpha_{1}\ln\left(\alpha_{1}\right)+\alpha_{2}\ln\left(\alpha_{2}\right)+\alpha_{3}\ln\left(\alpha_{3}\right)\right)$$

at the given scale k [38]. Multiple optimal scales can be determined by varying the scale parameter k within the candidate scales and selecting the scales that yield the first m smallest eigen-entropies, where m is a user-defined number. In comparison, the optimal scale method chooses the scale that yields the minimum eigen-entropy, and the multi-scale method just randomly chooses multiple fixed scales from the candidate scales for all points.

### 3.2. Features Extraction

We calculated 12 local 3D and 2D geometrical features (Table 2) for a point $q_{i}$ based on its k nearest neighbors. The 2D geometrical features are calculated from projected points on the XY plane. According to the experimental results of Wang et al. [16], higher separation accuracy can be achieved when more geometric features are used. However, when the number of geometric features surpasses a certain threshold, the improvement in the separation accuracy will reach diminishing returns. In addition, more computation time and memory are required when more geometric features are used. In related works, Ma et al. [14] used three geometric features; Yun et al. [24] used nine geometric features; Zhu et al. [26] used seven radiometric features and six geometric features; Wang et al. [16] used 26 geometric features and Wang et al. [15] used 32 geometric features. The above studies showed that no more than 10 geometric features are needed to stabilize the separation accuracy in leaf and wood separation studies [16]. We simply combine the 12 local 3D and 2D geometrical features over multiple scales for the multi-optimal-scale and the multi-scale methods to train models. Such a combination method is also used in Brodu et al. [29] and Wang et al. [36].

### 3.3. Separation Method

We use the random forest method to separate leaf and wood points in this study, as suggested in Wang et al. [16] and Weinmann et al. [31]. The random forest is a decision-tree-based ensemble learning method that was proposed in Breiman [39]. The learned model is a collection of weak models. Multiple decision trees are grown on random subsets of training data. The class determination is based on a majority vote fashion. Compared with other methods, it can handle high data dimensionality with highly correlated features and is fast and insensitive to overfitting. The random forest has three key parameters: the number of decision trees $n_{tree}$, the number of input features $n_{feature}$ used at each node, and the minimum leaf size $m_{leaf}$. The model accuracy increases when $n_{tree}$ increases until it reaches diminishing returns [15,16,26,31,32,33]. Increasing $n_{feature}$ improves the performance but may decrease the diversity of individual trees. A smaller $m_{leaf}$ makes the model more prone to capturing noise in training data.

### 3.4. Optimization Strategy

Compared with the optimal scale method, our proposed multi-optimal-scale method needs to calculate geometric features at multiple scales, which entails more calculation. To reduce the running time of our method, two strategies are employed. The first strategy is to compute the geometric features on a random sub-sampling of the tree point cloud called *core points* and conduct the separation of leaf and wood on the *core points*. Each point of the whole tree is then given the label of the nearest *core point*. The second strategy is to use parallel computation to divide the whole computation into several parts and process them in different central processing units to improve computation speed. The two strategies are commonly employed to reduce the computation time in the literatures [15,29,33]. We use the Parallel Computing Toolbox of MATLAB R2018b to parallel our method.

### 3.5. Evaluation

The performances of our multi-optimal-scale method, the multi-scale method, and the optimal scale method are evaluated using the accuracy statistical index [15,16,26]. The accuracy index is given by

(2) Accuracy=(Tw+Tl)/(w+l)

where w and l are the number of wood points and leaf points; Tw and Tl are the correctly identified wood points and leaf points, respectively.

The total running time includes the time used by scale selection, feature extraction, classifier training, and leaf and wood separation. For the multi-optimal-scale method, the speedup ratio is used to assess the efficiency of optimization strategy [12]. The speedup ratio of method a over method b is defined by

(3) Speedup ratio=Tb/Ta

where Ta and Tb are the total running time of the two methods, respectively.

## 4. Experimental Results and Discussion

Our experiments are conducted on a 64-bit Windows 7 with an Intel(R) Xeon(R) E5-2609 v4 1.7 GHz processor and 32GB RAM. The source code of our method is written in MATLAB programing language. Our implementation builds on the source code of Weinmann et al. [31,40]. In our experiments, the candidate scales form an arithmetic sequence. We vary k from 10 to 100, with a step size of 10. Varying k with a fixed step size to select a series of scales is commonly practiced by researchers [31,32,33,34]. In total, we have 10 candidate scales. For our multi-optimal-scale method, the number of optimal scales is varied from 2 to 10. In total, we have 9 multi-optimal-scale models. It is worth noting that for any given number of optimal scales, each point may have different sets of optimal scales. For the multi-scale method, the number of scales is also varied from 2 to 10. For each given number of scales, we train 50 multi-scale models and obtain the worst, best, and mean accuracy of the 50 models for each tree. For the random forest classifier, we set $n_{tree}=100$, $n_{feature}=sqrt\left(m\right)$, where m denotes the number of input features, as suggested in Breiman [39]. We set $m_{leaf}=10$ to avoid overfitting and obtain higher separation accuracy. The random forest classifier module of MATLAB is used to conduct the classification. About 10% of the *core points* for each tree is selected as training points to train the models with the simple random sampling method and the remaining *core points* are used for evaluating the accuracies.

**Figure 2:**
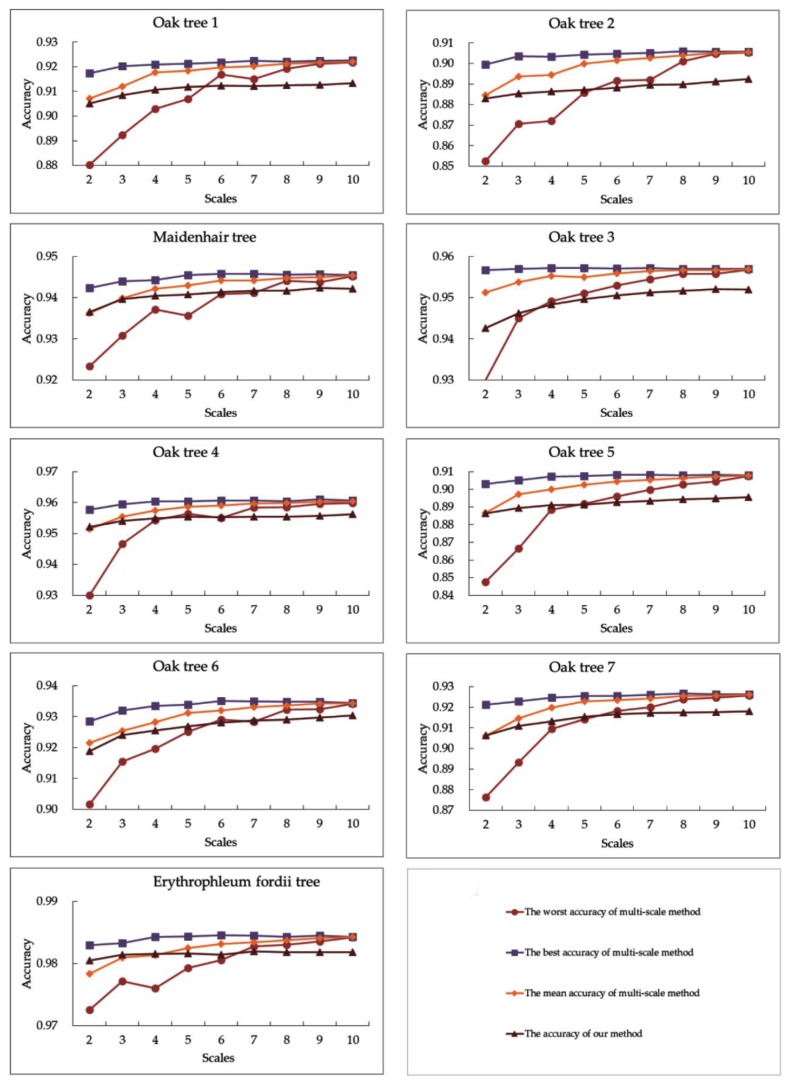
The worst, best, and mean separation accuracy of the multi-scale method and the separation accuracy of our method for each tree.

The experiment results are shown in Table 3 and Figure 2. We observe that the highest accuracy of the 9 multi-optimal-scale models are greater than that of the optimal scale model by about 1%–3% for each tree. For example, our method achieves the highest accuracy of 91.81% on the *Oak* tree 7. In comparison, the optimal scale method achieves an accuracy of 89.04%. The improvement in accuracy by our multi-optimal-scale method over the optimal scale method is of a similar size to those reported in the literature. For example, Weinmann et al. [31] showed that the separation accuracy of the optimal scale method is higher than the highest separation accuracy of the fixed scale method with random forest model by about 0.71% in Oakland dataset. For vegetation class, Brodu et al. [29] showed that the balanced accuracy of the multi-scale method was higher than the highest balanced accuracy of the fixed scale method with linear discriminant analysis model by about 0.68% in the Otira River scene. The separation accuracy of our method is more stable than the multi-scale method when the number of scales is no more than five. The multi-scale method tends to achieve higher accuracy when the scale number is greater than five. This is probably because our method captures the distinctive characteristics of leaf and wood points with fewer scales and the multi-scale method tends to get more distinctive information with more scales.

**Figure 3:**
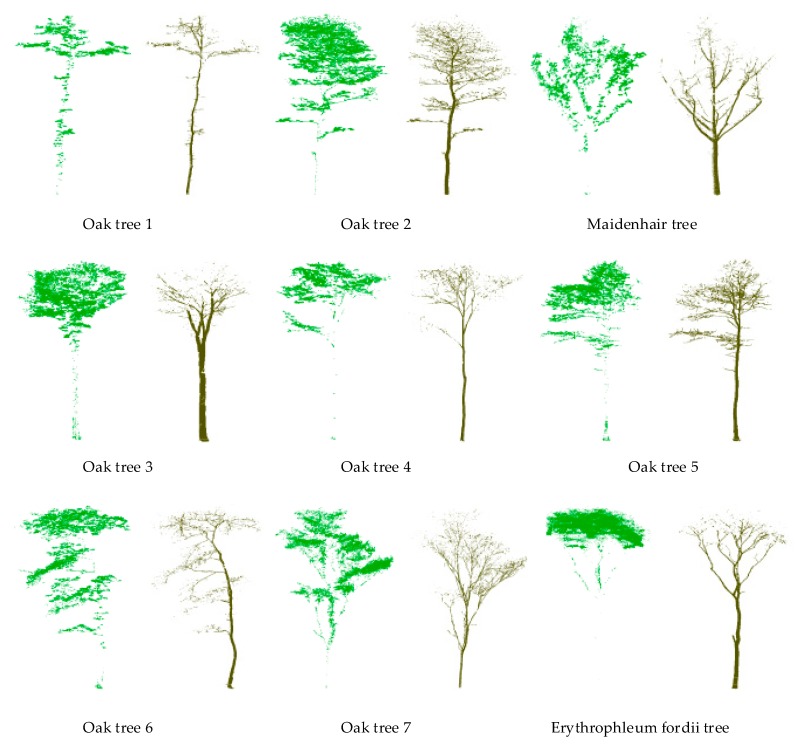
The separation results of our proposed multi-optimal-scale method with five optimal scales.

The separated leaf and wood points of our proposed multi-optimal-scale method with five scales for each tree are shown in Figure 3. It is worth noting that some small branch sections inside the canopy are misclassified as leaf points and some leaf points on the canopy surface are wrongfully labeled as wood points. Nevertheless, based on visual assessment, the overwhelming majority of the branches inside the canopy are separated from leaves successfully.

**Figure 4:**
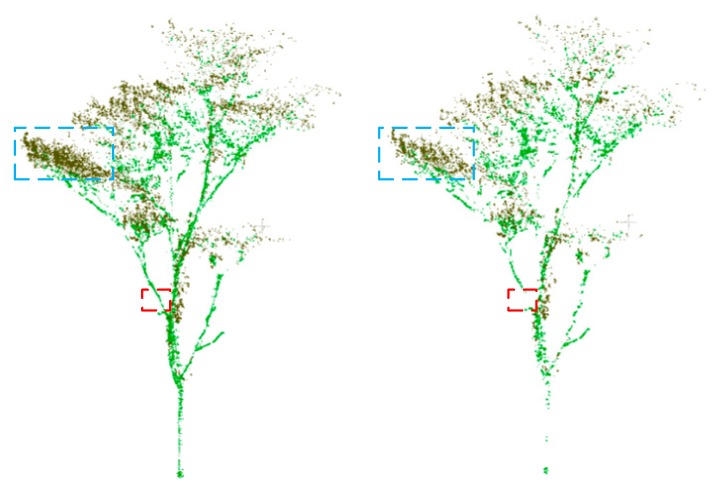
The incorrectly separated points of the optimal scale method (**left**) and our proposed multi-optimal-scale method with five scales (**right**). The two red boxes show that our proposed multi-optimal-scale method increases the number of correctly separated wood points. The two blue boxes show that our proposed multi-optimal-scale method decreases the number of incorrectly separated leaf points.

While our proposed multi-optimal-scale method only improves the separation accuracy by about 3% against the optimal scale method, our method greatly increases the separation accuracy for small branches and might facilitate the extraction of accurate above ground biomass and leaf area index [6,24,41]. To visually demonstrate the potential of our method in the extraction of the two high-level tree parameters, we plot the incorrectly separated points of *Oak* tree 7 of the optimal scale method and our proposed multi-optimal-scale method with five optimal scales in Figure 4 (the correctly separated points are not shown). Our method decreases the number of small branch sections that are misclassified as leaf points and may improve the estimation accuracy of above ground biomass (red box). Our method increases the number of correctly separated leaf points, which may be helpful to improve the estimation accuracy of dried leaf area index (blue box).

In Table 4, we list the running times of our multi-optimal-scale method with/without the optimization described in Section 3.4 when the number of scales is equal to five. In our experiments, we use 12 cores to accelerate our method. Our method has the highest speedup ratio of 60.04 for the Oak tree 2 and the lowest speedup ratio of 46.11 for the Maidenhair tree. The mean speedup ratio for all trees are up to 55.05. Clearly, the optimization greatly reduces the computation load of our method and makes it more amenable to larger datasets.

## 5. Conclusions

In this study, we propose a multi-optimal-scale method to separate leaf and wood points in tree point clouds. We propose a method to select multiple optimal scales among a series of scales for each point in the tree point cloud, which increases the distinctiveness of derived geometric features. The selected 3D and 2D features are extracted at the multiple optimal scales and the random forest model is used as the classifier to separate the leaf and wood points. Compared with the optimal scale method, our method achieves higher separation accuracy. Compared with the multi-scale method, our method achieves more stable accuracy with a limited number of optimal scales. The running time of our method can be greatly reduced when the optimization is introduced, making it applicable to large scenes.

## Figures and Tables

**Table 1:** The details of each tree point cloud.

Trees	Leaf Points	Wood Points	Average Point Density (mm)	Tree Height (m)	Core Points
Oak tree 1	2,122,328	1,220,188	0.9888	11.1069	334,252
Oak tree 2	7,429,900	4,924,099	0.9418	16.7122	1,235,400
Maidenhair tree	120,530	100,132	1.1586	1.4043	22,066
Oak tree 3	3,933,295	6,378,470	1.1421	24.0598	1,031,177
Oak tree 4	2,097,385	2,154,306	1.1458	11.3181	425,169
Oak tree 5	2,416,849	8,072,313	0.9334	21.6634	1,048,916
Oak tree 6	2,750,571	2,002,032	1.1095	10.9939	475,260
Oak tree 7	3,793,240	2,128,051	0.8768	7.7147	592,129
Erythrophleum fordii tree	2,061,679	1,806,859	4.9382	21.2738	386,854

**Table 2:** Geometrical features extracted from the tree point cloud. EV3D and EV2D denote the eigenvalue (sorted in ascend manner) and NV3D is the normal vector.

Feature	Description
Linearity3D	EV3D_3_/(EV3D_1_ + EV3D_2_ + EV3D_3_)
Planarity3D	EV3D_2_/(EV3D_1_ + EV3D_2_ + EV3D_3_)
Omnivariance3D	(EV3D_1_ × EV3D_2_ × EV3D_3_)^1/3^
Anisotropy3D	(EV3D_3_ − EV3D_1_)/EV3D_3_
Verticality3D	NV3Dz
Radius3D	Radius of 3D local neighborhood.
Density3D	Point density of 3D local neighborhood.
Zdiff3D	Height difference of 3D local neighborhood.
StdZ3D	Standard deviation of heights of 3D local neighborhood.
Radius2D	Radius of 2D local neighborhood.
Density2D	Point density of 2D local neighborhood.
Linearity2D	EV2D_2_/(EV2D_1_ + EV2D_2_)

**Table 3:** The separation accuracy of the optimal scale method and the highest separation accuracy of our proposed multi-optimal-scale method for each tree.

Trees	Optimal Scale Method	Our Method
Oak tree 1	0.8947	0.9133
Oak tree 1	0.8738	0.8923
Maidenhair tree	0.9194	0.9374
Oak tree 3	0.9301	0.9521
Oak tree 4	0.9452	0.9561
Oak tree 5	0.8772	0.8955
Oak tree 6	0.9058	0.9304
Oak tree 7	0.8904	0.9181
Erythrophleum fordii tree	0.9759	0.9820

**Table 4:** The running times of our multi-optimal-scale method with/without the optimization and the derived speedup ratio for each tree.

Trees	With Optimization (s)	Without Optimization (s)	Speedup Ratio
Oak tree 1	32.72	1714.80	52.41
Oak tree 1	90.37	5425.74	60.04
Maidenhair tree	10.04	462.92	46.11
Oak tree 3	74.41	4431.73	59.56
Oak tree 4	40.95	2244.31	54.81
Oak tree 5	74.98	4494.36	59.94
Oak tree 6	42.63	2224.73	52.18
Oak tree 7	49.75	2760.86	55.50
Erythrophleum fordii tree	36.49	2004.13	54.93
